# Correlation Analysis of Large-Span Cable-Stayed Bridge Structural Frequencies with Environmental Factors Based on Support Vector Regression

**DOI:** 10.3390/s23239442

**Published:** 2023-11-27

**Authors:** Jingye Xu, Tugang Xiao, Yu Liu, Yu Hong, Qianhui Pu, Xuguang Wen

**Affiliations:** 1 School of Civil Engineering, Southwest Jiaotong University, Chengdu 610031, China; 248515917@my.swjtu.edu.cn (J.X.); xiaotg@my.swjtu.edu.cn (T.X.); 15923082927@126.com (Y.L.); qhpu@vip.163.com (Q.P.); 2 Guangxi Key Laboratory of International Join for China-ASEAN Comprehensive Transportation, Nanning University, Nanning, Guangxi, 530000, China; xuguang1959@163.com

**Keywords:** structural health monitoring, environmental temperature, operation modal analysis, stochastic subspace identification algorithm, environmental humidity, SVR

## Abstract

The dynamic characteristics of bridge structures are influenced by various environmental factors, and exploring the impact of environmental temperature and humidity on structural modal parameters is of great significance for structural health assessment. This paper utilized the Covariance-Driven Stochastic Subspace Identification method (SSI-COV) and clustering algorithms to identify modal frequencies from four months of acceleration data collected from the health monitoring system of the Jintang Hantan Twin-Island Bridge. Furthermore, a correlation analysis is conducted to examine the relationship between higher-order frequency and environmental factors, including temperature and humidity. Subsequently, a Support Vector Machine Regression (SVR) model is employed to analyze the effects of environmental temperature on structural modal frequencies. This study has obtained the following conclusions: 1. Correlation analysis revealed that temperature is the primary influencing factor in frequency variations. Frequency exhibited a strong linear correlation with temperature and little correlation with humidity. 2. SVR regression analysis was performed on frequency and temperature, and an evaluation of the fitting residuals was conducted. The model effectively fit the sample data and provided reliable predictive results. 3. The original structural frequencies underwent smoothing, eliminating the influence of temperature-induced frequency data generated by the SVR model. After eliminating the temperature effects, the fluctuations in frequency within a 24 h period significantly decreased. The data presented in this paper can serve as a reference for further health assessments of similar bridge structures.

## 1. Introduction

With the continuous increase in the number and scale of transportation infrastructure, there is a growing demand for regular inspection and maintenance to ensure its long-term stable operation. Through long-term monitoring of bridge structures, real-time monitoring of the structures can be achieved, aiding in predicting the lifespan of the structures and formulating rational maintenance plans. Simultaneously, the long-term data collected by structural health monitoring (SHM) systems can provide robust support for data-driven decision-making. Combining advanced data analysis techniques, engineers can develop more precise maintenance strategies and engineering plans, enabling the more efficient allocation of resources and enhancing overall operational efficiency.

In SHM, changes in the dynamic characteristics of structures are crucial for assessing structural health. Modal frequency variations are among the key parameters used to evaluate structural conditions [1,2,3,4,5]. However, the modal frequencies of real engineering structures are influenced by numerous environmental factors, such as temperature, humidity, ambient noise, and wind, among others. Changes in modal parameters induced by environmental factors are often significant and can even exceed those caused by structural damage or operational loads [6,7,8,9]. Generally, temperature is recognized as the primary environmental factor affecting structural frequencies [6]. In recent years, numerous researchers have undertaken studies to assess the impact of temperature on the modal parameters of bridge structures and investigate methods to mitigate its influence.

Several researchers have investigated the reasons behind the influence of temperature changes on structural modal frequencies. Ni et al. [10], based on field-measured acceleration and temperature data from the Sutong Bridge, studied the variations in natural frequencies and damping ratios with temperature and vibration levels. Their research revealed that the natural frequencies of bridge structures decrease with increasing temperature, possibly due to changes in the elastic modulus of construction materials induced by temperature fluctuations. Cho and Cho [11] conducted a long-term monitoring study on the dynamic characteristics of two short-span concrete bridges and found that the natural frequencies of the bridges decrease with rising temperatures. They validated this modal change through model updating, attributing it to the temperature-dependent elasticity modulus of concrete. Similar conclusions were drawn by Zhou and Sun [12], who combined environmental factors with six years of modal frequency data from the East Sea Bridge. They identified cyclic variations in frequency and observed that all modal frequencies of the structure decrease with increasing temperature due to changes in the elastic modulus of structural materials. Cai et al. [13] analyzed the impact of temperature on the free vibration characteristics of simply supported beams and theoretically deduced that the influence of temperature on the natural frequency of simply supported beams is primarily caused by changes in the elastic modulus of the beam material, with the structural dimensions having a less significant impact. Anastasopoulos et al. [14] analyzed temperature fluctuations during monitoring periods using modal data collected from a steel arch railway bridge equipped with Fiber Bragg Grating Strain Sensors. They found that temperature can induce changes in the bridge’s overall stiffness, leading to variations in natural frequencies. Sun et al. [15] studied the relationship between modal frequencies and temperature in a controlled temperature chamber for concrete continuous beam bridge models and steel cable-stayed bridges. They observed that the influence of temperature on structural frequencies is closely related to boundary conditions and that temperature may alter the bridge’s boundary conditions.

Different viewpoints exist among scholars regarding the pattern of modal frequencies decreasing with rising environmental temperatures. Mosavi et al. [16] investigated the relationship between modal characteristics and temperature in a two-span steel-concrete composite bridge. They found that during the noon hours, the first five frequencies of the main beam were higher than those during the night, with three of the frequencies positively correlated with temperature. A similar phenomenon was observed at the Saint Michel Bridge [17]. Michal and Katarína [18] used the Random Subspace Method to identify acceleration data from a steel bridge and processed it in conjunction with temperature data. They observed that the first two frequency modes of the structure increased with rising temperature, and some frequencies showed non-linearity when fitted with linear regression functions against temperature data.

Research has shown that eliminating the influence of a single environmental temperature can provide a more accurate assessment of bridge conditions [19,20]. The performance of temperature effects in the presence of multiple environmental factors has also received attention. Mao et al. [21] employed the Hilbert–Huang Transform method to identify continuous modals over a year for the Sutong Bridge. They analyzed the impact of environmental temperature and traffic volume on modal frequencies and found that temperature effects outweighed traffic volume, being the most significant factor for vertical and torsional modal frequencies. Yang et al. [22] conducted statistical characterization and probabilistic prediction of environmental load data (wind speed, wind direction, temperature, and humidity) and acceleration response data collected by the SHM system of the Jiashao Multi-Tower Cable-Stayed Bridge. They established mapping relationships between specific load sources and structural responses. Chu et al. [1] proposed a time-series decomposition method to divide the time series of structural dynamic characteristics into different time scales and explore their relationship with periodic environmental loads. Their research indicated that an increase in temperature leads to a decrease in modal frequencies and damping ratios, while modal frequencies and damping ratios show no significant correlation with humidity. He et al. [23] considered the influence of temperature and humidity on frequency monitoring data and developed a temperature and humidity elimination method based on BP neural networks, improving the accuracy of bridge condition reliability assessment. He et al. [24] established a “Frequency-Temperature-Humidity” long-term equilibrium model based on cointegration theory for monitoring data from a three-span concrete bridge model. This model can be used to predict the trend of frequency changes and eliminate the influence of temperature and humidity on frequency. Mu et al. [25] developed a Bayesian network algorithm to identify the relationship between modal frequencies and various environmental factors. They found that considering the correlation between temperature and relative humidity improved the prediction accuracy when predicting modal frequencies. Meng and Zhu [26] quantitatively compared the influence of temperature variations, temperature gradients, and material properties on the dynamic performance of large-span suspension bridges through multiscale modeling. They discovered that the time-varying dynamic characteristics of large-span suspension bridges are primarily induced by thermal stress-hardening effects. Wang et al. [27,28] proposed an EFR model based on multi-order frequencies and a local thermal-frequency correlation model incorporating Gaussian mixtures to eliminate the variability in modal frequencies caused by temperature, humidity, and wind speed. They verified the effectiveness of these two methods through experiments on cable-stayed bridges.

This study analyzes nearly five months of vibration acceleration and temperature and humidity data collected from four representative vertical acceleration sensors located inside the main beam of the Jintang Hantan Twin-Island Bridge in Chengdu, China, using the Covariance-Driven Stochastic Subspace Identification method (SSI-COV). It employs clustering algorithms to automatically identify modal frequencies from steady-state diagrams and utilizes Support Vector Machine Regression (SVR) analysis to model the identified frequencies in relation to environmental temperature and humidity. Finally, based on the fitted model, the influence of temperature on the identified frequencies is eliminated, and the post-temperature-adjusted frequency fluctuations are observed. Finally, the main conclusions of the article were summarized, and potential directions for further in-depth research in the future were discussed.

## 2. Hardware, Equipment, and Methods

### 2.1. Bridge Monitoring System Description

**Figure 1:**
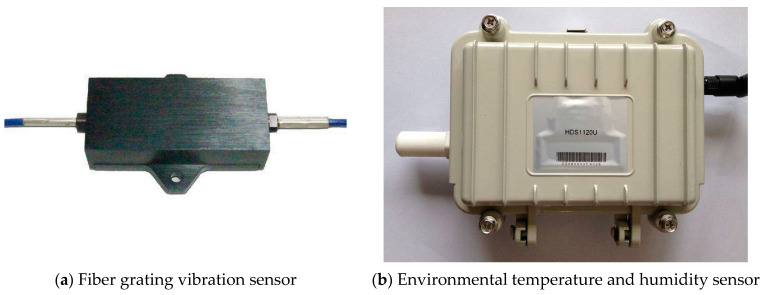
Fiber brag grating vibration sensor and environmental temperature and humidity sensor.

**Figure 2:**
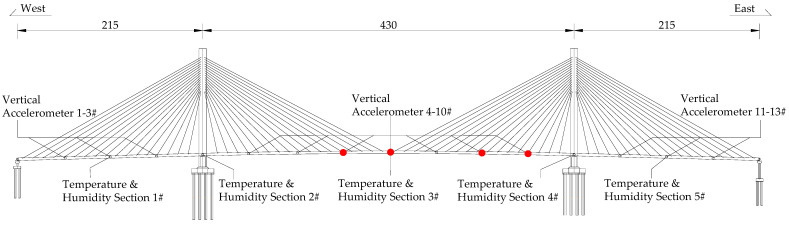
Location of accelerometers and temperature/humidity sensors.

The Jintang Hantan Twin-Island Bridge, located in Jintang County, Chengdu City, Sichuan Province, China, has a total length of 860 m, featuring a primary span of 430 m, and is constructed as a steel box girder cable-stayed bridge. To enable real-time monitoring of the bridge’s operational condition, a health monitoring system has been installed on the main beam of the cable-stayed bridge. The sensors related to acceleration and environmental temperature and humidity data in the monitoring system include 13 vertical acceleration measurement points (with a total of 26 acquisition channels) and 5 temperature and humidity sections (each section containing 7 temperature and humidity sensors). The fiber Bragg grating vibration sensor is used to collect acceleration data, while the environmental temperature and humidity sensor are employed for collecting temperature and humidity data. The images and detailed parameters of both sensors are shown in Figure 1 and Table 1 and Table 2. The distribution of monitoring points across the entire bridge is depicted in Figure 2. Due to hardware-related issues with certain sensors, their data output is not stable. While ensuring the accuracy and effectiveness of frequency identification, this paper selected data collected from vertical accelerometers at four locations on the main span (No. 6, 7, 9, and 10), in addition to data from temperature and humidity sensors at 5 cross-sections spanning the entire bridge.

### 2.2. Automated Covariance-Driven Stochastic Subspace Identification Method (SSI-COV) with the Clustering Algorithm

The automated Covariance-Driven Stochastic Subspace Identification method (SSI-COV) was developed by Magalhães et al. [29], and the incorporation of clustering algorithms was inspired by Yonggang [30] and Eric [31]. The Stochastic Subspace Method [32] is a time-domain approach that effectively mitigates data distortion during time-frequency conversion. If there are l output channels, with r of them being reference channels, the output data are used to construct a (2i) row × j column Hankel matrix, which is then decomposed into ‘past’ and ‘future’ segments, as shown in Equation (1):

(1) $$H=\frac{1}{\sqrt{j}}\left(\frac{\begin{array}{cccc} y_{0}^{ref} & y_{1}^{ref} & \cdots & y_{j-1}^{ref}\\ y_{1}^{ref} & y_{2}^{ref} & \cdots & y_{j}^{ref}\\ \vdots & \vdots & \ddots & \vdots \\ y_{i-1}^{ref} & y_{i}^{ref} & \cdots & y_{i+j-1}^{ref} \end{array}}{\begin{array}{cccc} y_{i} & y_{i+1} & \cdots & y_{i+j-1}\\ y_{i+1} & y_{i+2} & \cdots & y_{i+j}\\ \vdots & \vdots & \ddots & \vdots \\ y_{2i-1} & y_{2i} & \cdots & y_{2i+j-2} \end{array}}\right)=\frac{Y_{p}^{ref}}{Y_{f}}$$

In which $y_{k}^{ref}\in R^{r}$, $y_{k}\in R^{l}$, i represents the number of rows in the Hankel matrix, and j represents the number of columns in the matrix. If all s output data are used for analysis, then s=2i+j−1. Subsequently, a Toeplitz matrix is constructed using the Hankel matrix, as shown in Equation (2):

(2) $$T=Y_{f}Y_{p}^{refT}\left(\begin{array}{cccc} R_{i} & R_{i-1} & \cdots & R_{1}\\ R_{i+1} & R_{i} & \cdots & R_{2}\\ \vdots & \vdots & \ddots & \vdots \\ R_{2i-1} & R_{2i-2} & \cdots & R_{i} \end{array}\right)$$

**Figure 3:**
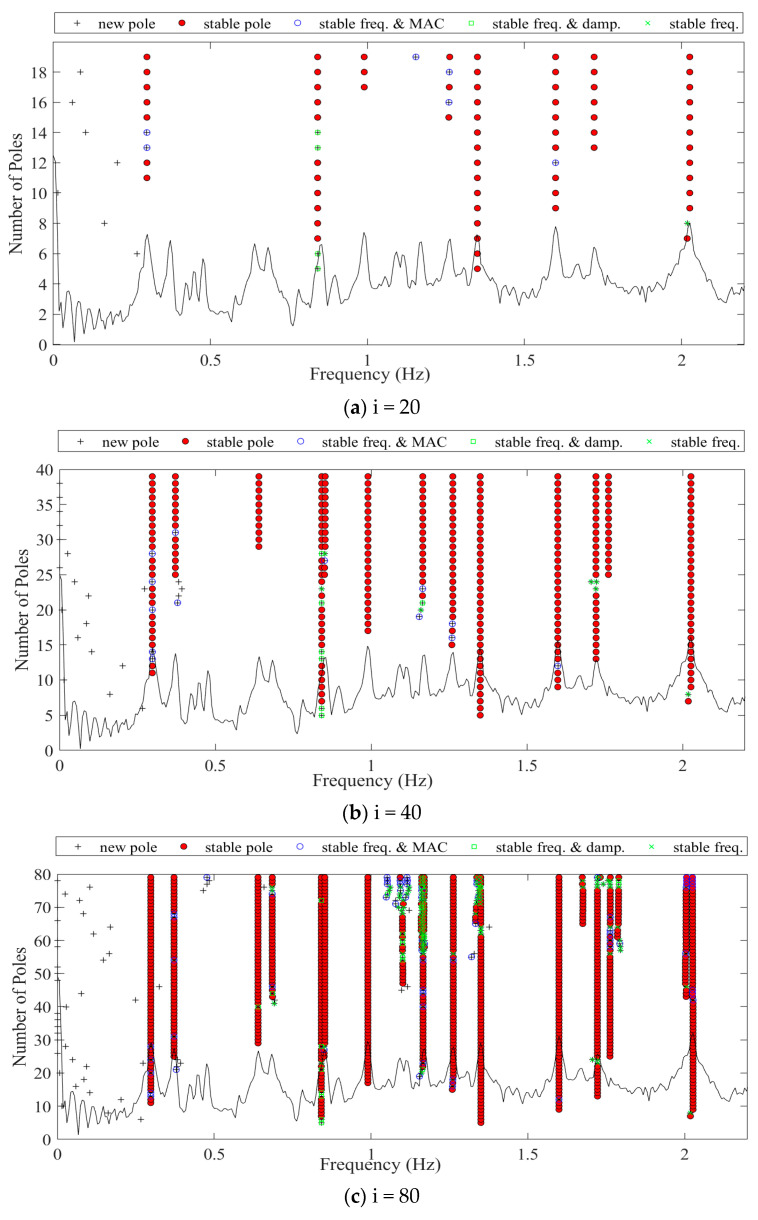
Stability diagrams of auto SSI-COV with the Clustering Algorithm.

The Toeplitz matrix is a li×ri dimensional matrix; therefore, the control parameter i will affect the size of the T matrix, and the scale of the T matrix significantly impacts the identification results. After obtaining the Toeplitz matrix, identification frequencies can be obtained through processes such as singular value decomposition and system order determination. To demonstrate the algorithm’s workflow and identification performance, this paper extracted acceleration data from the cable-stayed bridge recorded on 28 July from 02:00 to 02:10. The automated SSI-COV method with the Clustering Algorithm was used to identify the bridge frequencies under different control parameters. The results are shown in Table 3 and Figure 3. Table 3 displays the numerical solutions of modal frequencies obtained for the cable-stayed bridge using finite element analysis. It also provides the results of the automated SSI-COV method for the first 10 mode frequencies identified at different values of the control parameter i, along with the associated computation times. It can be observed that as the control parameter i and the Toeplitz matrix size increase, the number of identified frequencies also increases. However, at the same time, the number of false modes identified and the computational time required significantly increased. In this study, for a comprehensive balance between computational accuracy and efficiency, we chose i = 20 for identification.

## 3. Identification Results

### 3.1. Subsection

The automated SSI-COV method was employed to analyze the acceleration data at 10 min intervals from April to August 2023 for the cable-stayed bridge. After removing outliers and missing data, the method successfully identified the first six significant structural frequencies, as shown in Table 4. It can be observed that each frequency exhibits varying degrees of fluctuation, with the maximum relative change being 4.2%. Additionally, the fluctuation magnitude decreases as the mode order increases for the first five modes.

### 3.2. Correlation Research between Temperature, Humidity and Frequency

In this study, the time intervals for temperature and frequency identification were both set at 10 min. Due to partial sensor data loss and errors, presenting the accelerometer data for the entire five months would be challenging as it would be hindered by significant interruptions in the dataset, making it difficult to observe patterns effectively. Therefore, the accelerometer data from the week with the highest data volume in July was chosen for identification and analysis. The synchronized time series of measured frequency and temperature can be computed as shown in Equation (3).

(3) $$\left.\begin{array}{l} F^{c}=(f_{1}^{c},f_{2}^{c},\cdots f_{m}^{c},\cdots f_{M}^{c})\\ T=(t_{1},t_{2},\cdots t_{m},\cdots t_{M}) \end{array}\right\}$$

where $f_{m}^{c}$ and $t_{m}$ represent the frequency and temperature data for the m-th 10 min interval, respectively, with the superscript c denoting the frequency order.

**Figure 4:**
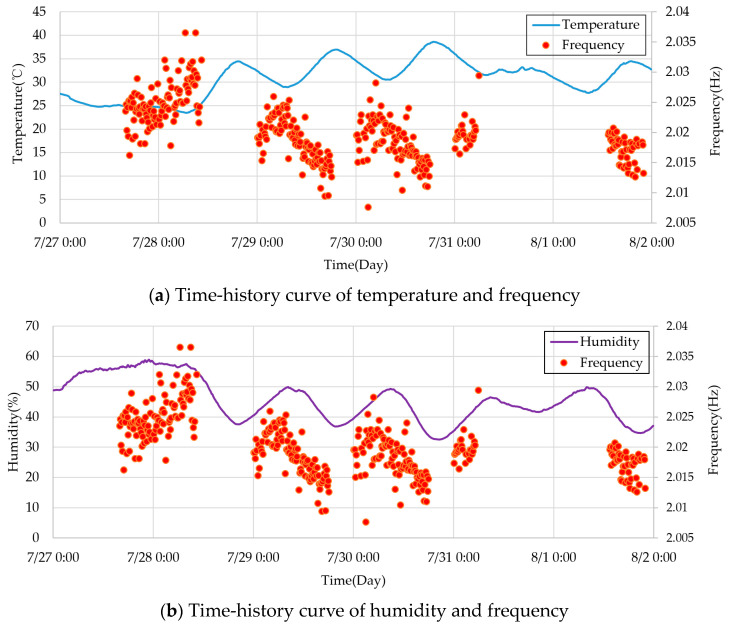
Time-history curve of temperature and frequency from 27 July to 2 August 2023.

**Figure 5:**
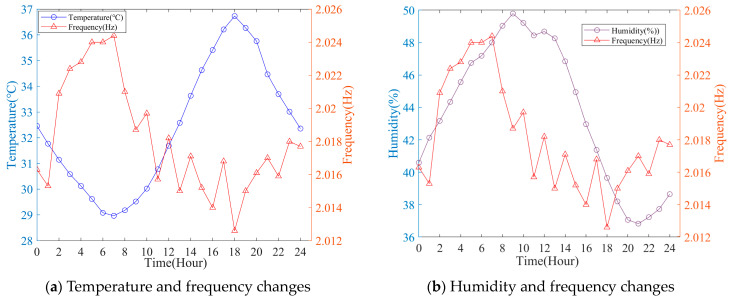
No. 6: Modal frequency variation with the influence of temperature and humidity.

The time history curves of temperature, humidity, and the 6th-order frequency of the cable-stayed bridge from 27 July to 2 August are shown in Figure 4. From the figure, it can be observed that temperature, humidity, and frequency all exhibit relatively regular fluctuations on a daily basis. To better illustrate the temperature, humidity, and frequency variation patterns, Figure 5 presents the curves of temperature and 6th-order frequency, as well as humidity and 6th-order frequency for 29 July. It is evident from the figure that the modal frequency of the structure significantly decreases with increasing temperature.

**Figure 6:**
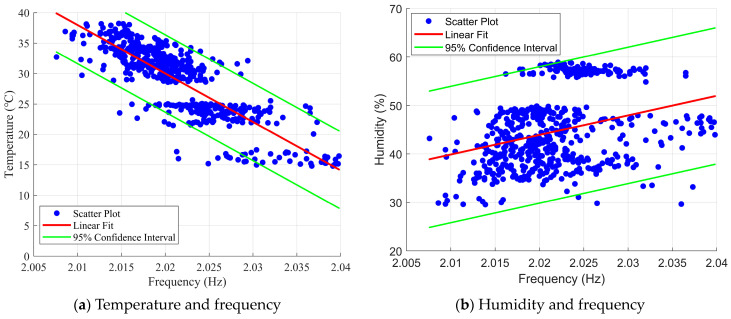
Distributions of No. 6 modal frequencies and the linear regressions respective to temperature and humidity.

To further investigate the correlation between temperature and frequency, a linear fit was applied to the temperature and 6th-order frequency data from April to July. The fitting results are shown in Figure 6. The correlation coefficient between the 6th mode identification frequency and temperature from April to July was −0.8283, while the correlation coefficient with humidity was 0.3209. Combined with a 95% confidence interval, it can be observed that the temperature-frequency data are concentrated on both sides of the fitted curve. This indicates a strong negative correlation between the modal frequency of the Jintang Hantan Twin Island Bridge and temperature, while there is no significant linear correlation between humidity and frequency.

## 4. Temperature-Frequency Nonlinear Model Analysis

The Support Vector Machine (SVR) regression model was employed to analyze the relationship between modal frequency and environmental temperature. Since humidity was found to have a minor impact on modal frequencies through correlation analysis in the previous sections, this study only established a model for modal frequencies and environmental temperature, excluding humidity as a predictor. It should be noted that, due to the limited sample data collected, the model established in this study is only applicable to the range of normal atmospheric temperature variations. The data used were collected during the spring and summer seasons in Chengdu, with temperature variations ranging from 14.8 to 38.2 °C. Effective monitoring data from April to August (i.e., no missing data for frequency and temperature) were selected for this study. The total number of collected samples amounted to 547 sets. Following a ratio of 0.85:0.15, the collected samples were randomly divided into training and testing sets. Among these, 465 sets of data were selected as training samples to build the model, denoted as $f_{train}$ and $t_{train}$. Subsequently, 82 sets of data were employed as testing samples to assess and verify the predictive capability of the model, labeled as $f_{test}$ and $t_{test}$.

### 4.1. Nonlinear Model Based on SVR Regression Analysis

Support Vector Regression (SVR) is a non-linear regression analysis method derived from Support Vector Machine (SVM) technology. The core of SVR lies in mapping complex non-linear problems to a new linear space using a kernel function. The linear equation in the new space can be expressed as follows:

(4) $$y(x)=w^{T}\varphi (x)+b+e$$

where w is the weight vector, b is a constant, e represents the error, and φ(x) is the transformation function with respect to the input vector x. If there is a training sample $\left\{x_{i},y_{i}\right\}(i=1,2,\cdots ,N)$, then the SVR model can be transformed into an optimization problem for solving [33]. This results in a system of linear equations,

(5) $$\left[\begin{array}{cc} 0 & 1_{N\times 1}\\ 1_{1\times N} & \Omega +\gamma^{-1}I_{N} \end{array}\right]\left[\begin{array}{c} b\\ \alpha \end{array}\right]=\left[\begin{array}{c} 0\\ Y \end{array}\right]$$

where $Y={\left[y_{1},\cdots ,y_{N}\right]}^{T}$ and $\alpha ={\left[\alpha_{i},\cdots ,\alpha_{N}\right]}^{T}$ are Lagrange multipliers, γ is the regularization parameter. Ω is the kernel matrix computed based on the kernel function. Each element in Ω can be calculated using Equation (6),

(6) $$\Omega_{ij}=\varphi {\left(x_{i}\right)}^{T}\varphi (x_{j})=K(x_{i},x_{j})$$

Thus, by solving Equation (4), the required fitting model can be obtained. Observing Equation (5), it is not difficult to find that in the SVR method, the kernel matrix Ω is determined by the kernel function $K(x_{i},x_{j})$. Therefore, we do not need to know the exact expression of the transformation function φ(x); we only need to determine the kernel function $K(x_{i},x_{j})$ to determine the regression model. There are many types of kernel functions to choose from, depending on the specific circumstances. In this study, the commonly used Gaussian radial basis function (RBF) $K(x_{i},x_{j})=e^{-{\left\|x_{i}-x_{j}\right\|}^{2}/2\sigma^{2}}$ was selected for modeling and analysis.

**Figure 7:**
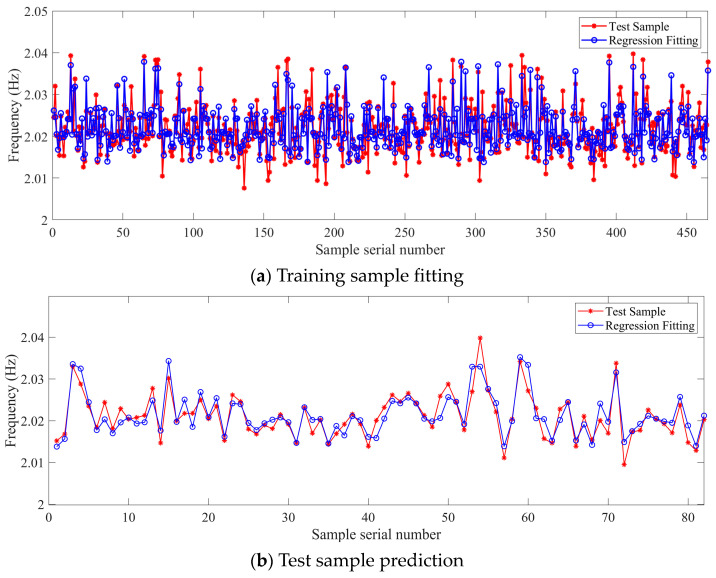
Regression of the 6th-order frequency based on the SVR nonlinear model.

**Figure 8:**
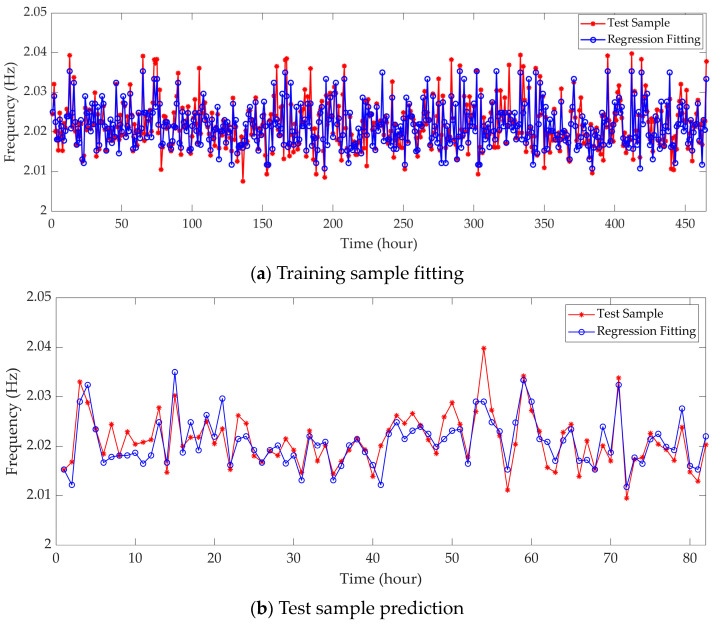
Regression of the 6th-order frequency based on a random forest model.

In this study, 6th-order recognition frequency was selected for investigation. The key model parameters of the SVR model are listed in Table 5, and the calculation results of the regression model are shown in Figure 7. It can be seen that both the fitting and prediction results closely match the curves of the measured samples. Therefore, it is evident that the SVR regression method based on the radial basis kernel function can also establish a reasonably effective model for the temperature-frequency relationship. As a contrast, the random forest model is also used to train and predict the same frequency data; the number of decision trees constructed is 10. The recognition results are shown in Figure 8. The accuracy of the random forest model will be discussed in a subsequent section.

### 4.2. Model Quality Analysis

To assess and compare the fitting quality of the models, an analysis of the model’s residuals was conducted. If the fitting model is denoted as $F^{c}(t_{h})$, then the fitting residuals are defined as $e_{f}^{c}(h)={\overset{⌣}{f}}_{h}^{c}-F^{c}({\overset{⌣}{t}}_{h})$, and the prediction residuals as $e_{p}^{c}(h)={\overset{⏜}{f}}_{h}^{c}-F^{c}({\overset{⏜}{t}}_{h})$. Based on the fitting residuals and prediction residuals, four evaluation metrics were calculated: root mean square error (RMSE), coefficient of determination (R^2^), kurtosis, and skewness. The root mean square error (RMSE) is the square root of the second sample moment of the difference between predicted values and observed values, reflecting the accuracy of the model. The kurtosis of the residuals is defined as:

(7) $$Kurt=\frac{\frac{1}{H}\sum_{h=1}^{H}{\left(e_{h}^{c}-{\bar{e}}^{c}\right)}^{4}}{{\left[\frac{1}{H}\sum_{h=1}^{H}{\left(e_{h}^{c}-{\bar{e}}^{c}\right)}^{2}\right]}^{2}}$$

The skewness of the residuals can be defined as,

(8) $$Skew=\frac{\frac{1}{H}\sum_{h=1}^{H}{\left(e_{h}^{c}-{\bar{e}}^{c}\right)}^{3}}{{\left[\frac{1}{H}\sum_{h=1}^{H}{\left(e_{h}^{c}-{\bar{e}}^{c}\right)}^{2}\right]}^{3/2}}$$

The root mean square error (RMSE) can be defined as,

(9) $$RMSE=\sqrt{\frac{1}{N}\sum_{h=1}^{N}{\left(F^{c}(t_{h})-\;f_{h}^{c}\right)}^{2}}$$

where N is the length of $f_{train}$ or $f_{test}$.

The coefficient of determination (R^2^) can be defined as,

(10) $$R^{2}=1-\frac{SS_{res}}{SS_{tot}}$$

where $SS_{res}=\sum_{h}e_{h}^{c}{}^{2}$ is the sum of squares of residuals, $SS_{tot}=\sum_{h}(F^{c}(t_{h})-{\bar{F}}^{c}(t_{h}){)}^{2}$ is the total sum of squares.

Root Mean Square Error (RMSE) reflects the accuracy of the model, and its value is always non-negative, with a value of 0 indicating a perfect fit to the data. In general, a lower RMSE is better than a higher one. The coefficient of determination (R^2^) is used to assess the goodness of fit of a regression model, with a value closer to 1 indicating a higher degree of fit. The kurtosis and skewness of residuals are both measures of the normality of their amplitude distribution. Kurtosis is used to assess the steepness of the residual distribution: Kurt<3 indicates that the residual distribution is flatter than the normal distribution, while Kurt>3 indicates that the residual distribution is sharper than the normal distribution. Skewness is used to evaluate the asymmetry of the residual distribution: Skew<0 indicates that the majority of residual values (including the median) are located to the right of the mean, while Skew>0 indicates that the majority of residual values (including the median) are located to the left of the mean. Therefore, when Kurt is closer to 3 and Skew is closer to 0, it indicates that the residual distribution is closer to normal. A residual distribution closer to normality indicates a higher model quality.

**Figure 9:**
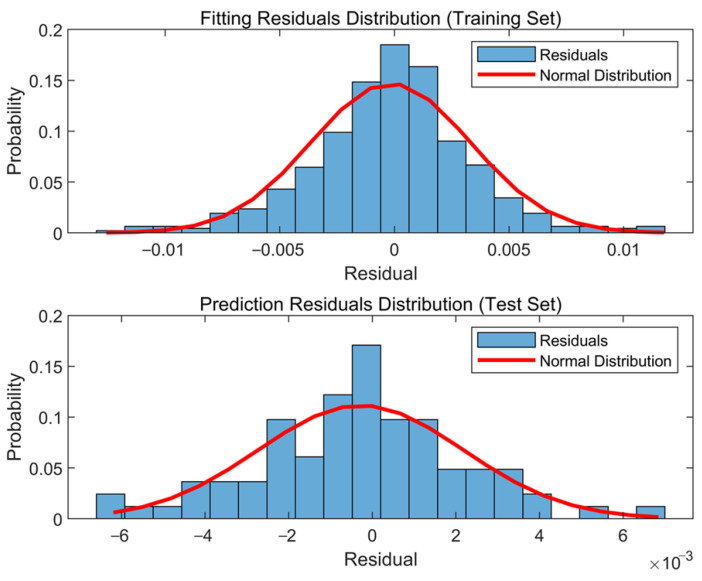
Residuals distribution of the 6th-order frequency based on the SVM model.

Figure 9 shows the residual distribution of the support vector machine model, and Table 6 shows the error evaluation parameters of the SVR model and random forest model for identifying the 6th-order frequency. Comparing the residual distribution with the normal distribution curve, it is evident that the distribution of fitting residuals is closer to normal than that of prediction residuals, and the parameters of the residual distribution perform relatively well. Both the coefficient of determination R^2^ for fitting and prediction are close to 1, and the root mean square error values are small. This indicates that the fitting and prediction have achieved a high level of accuracy, effectively reflecting the true state of the training samples. Compared to the SVR model, the Random Forest model performs better in terms of RMSE for fitting results. However, both the R^2^ for fitting results and the RMSE and R^2^ for prediction results are worse than those of the SVR model. This indicates that the SVR model demonstrates superior overall performance in both fitting and prediction.

### 4.3. Eliminate Temperature Effects

**Figure 10:**
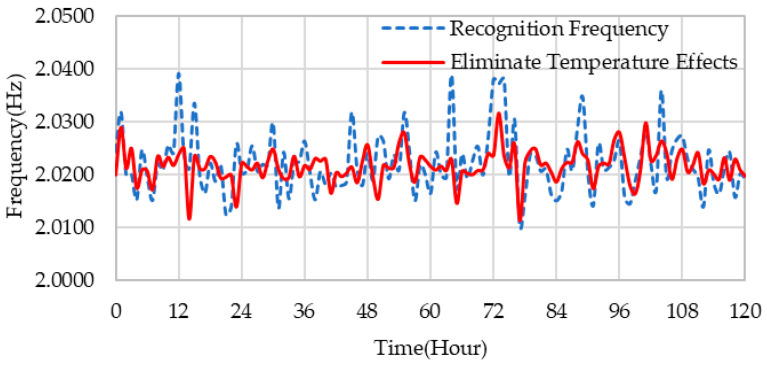
Frequency variation after the elimination of temperature influence.

To mitigate the influence of temperature on the identified frequencies, the temperature-frequency model proposed by Fan et al. [34] and validated on the Tingjiu Bridge was adopted. The formula for this model is shown in Equation (11).

(11) $$f_{h}^{c}={\bar{f}}^{c}+{\tilde{f}}_{h}^{c}-F^{c}(t_{h})$$

where ${\bar{f}}^{c}$ represents the expected value of the cth order frequency, and ${\tilde{f}}_{h}^{c}$ is the frequency value identified from measured data. Figure 10 shows the time history of the 6th-order frequency after eliminating the temperature effect. The frequency after eliminating the effect of temperature had a maximum change of 1.6% and an average change of 0.4% compared to the frequency before elimination. It can be observed that after removing the temperature influence, the frequency fluctuations with a 24 h periodicity have been significantly reduced. Therefore, this obtained frequency can better characterize changes in structural properties in structural health monitoring. Additionally, this result also demonstrates the excellent capability of the support vector machine nonlinear model in effectively modeling the relationship between modal frequency and environmental temperature.

## 5. Discussion and Conclusions

Based on four months of acceleration and temperature-humidity data from the Jintang Hantan Twin Island Bridge health monitoring system, this study successfully employed the Covariance-Driven Stochastic Subspace Identification (SSI-COV) method and clustering algorithm to identify acceleration data, obtaining stable higher-order frequencies. Subsequently, a correlation analysis was conducted to investigate the influence of environmental factors on these higher-order frequencies. It also employed Support Vector Machine Regression (SVR) for modeling analysis and analyzed the residuals of the SVR model. Finally, the real structural frequencies, free from environmental influences, were obtained. The specific conclusions are as follows:

1. Linear regression was used to observe the relationship between identified frequencies and environmental temperature. The results indicated a significant negative correlation between the bridge’s frequency and environmental temperature, while humidity showed no significant correlation with modal frequencies.
2. A nonlinear Support Vector Machine (SVM) model was employed for fitting and modeling. Residual analysis of the model revealed a clear linear relationship between frequency and temperature. The SVM model effectively fit the sample data and provided reliable predictive results.
3. After obtaining temperature-induced frequency data from the SVR model, the original structural frequencies were smoothed, and temperature influences were eliminated. It was observed that, after eliminating temperature effects, the frequency fluctuations with a 24 h period significantly decreased.

In this study, the influence of temperature and humidity on the modal frequencies of large-span cable-stayed bridges was analyzed, and the higher-order modal frequencies after eliminating the temperature influence were obtained. It can serve as a reference for the health monitoring of similar large-span cable-stayed bridge structures in practical engineering and can also serve as a foundation for further research on the impact of other environmental factors on bridge structural modal parameters.

Due to the complexity of the relationship between non-uniform temperature fields and structural frequencies and the limited number of existing bridge measurement points, this study only included uniform temperature effects without considering the non-uniform temperature field caused by structural spatial distribution and sunlight factors. Additionally, the impact of other environmental and operational factors (such as wind, vehicle loads, and noise interference) on the modal frequencies of the cable-stayed bridge should be addressed in future research phases.

## Figures and Tables

**Table 1:** Parameters of the fiber brag grating vibration sensor.

Parameters	Numerical Value
standard range	2 G
measurement precision	2‰ F.S.
resolution	0.1‰ F.S.
measurement frequency	10 HZ
natural frequency	>2 kHZ
sampling frequency	50 HZ

**Table 2:** Parameters of the temperature and humidity sensor.

Parameters		Numerical value
standard range	humidity	0~100% RH
	temperature	−20~+80 °C
measurement precision	humidity	±3% RH (11~89% RH, 25 °C)
	temperature	±0.5 °C (25 °C)
sampling frequency		≥0.02 Hz

**Table 3:** Numerical solution and identification results of the automated SSI-COV under different control parameter i.

		Identified Frequencies (Hz)										Computation Time (s)
No. Mode		1	2	3	4	5	6	7	8	9	10	
Finite Element		0.293	0.879	1.367	1.601	1.758	2.014	\	\	\	\	\
Automated SSI-COV	i = 20	0.298	0.841	1.350	1.599	1.722	2.026	\	\	\	\	36.40
	i = 40	0.298	0.640	0.841	0.990	1.166	1.262	1.350	1.599	1.722	1.762	39.53
	i = 80	0.298	0.372	0.640	0.685	0.842	0.853	0.990	1.166	1.262	1.350	43.28

**Table 4:** Identified frequencies (from April to August, 2023).

Parameters/No. Mode	Mean Value/Hz	Maximum Value/Hz	Minimum Value/Hz	Relative Difference/%
1	0.2977	0.3061	0.2940	4.2
2	0.8412	0.8585	0.8312	3.28
3	1.3480	1.3691	1.3303	2.92
4	1.5959	1.6100	1.5871	1.44
5	1.7602	1.7699	1.7501	1.13
6	2.0217	2.0398	2.0076	1.6

**Table 5:** The key model parameters of SVR model.

Parameters	Numerical Value
C	20
gamma	2
epsilon	0.01
tol	0.001

**Table 6:** Error parameters of regressive models.

Model	Error Type		Identification Frequency (Hz)
Support vector machine regression	Fitting	Root mean square error (RMSE)	0.0082823
		Coefficient of determination (R^2^)	0.82141
		Kurtosis	4.4081
		Skewness	−0.13324
	Prediction	Root mean square error (RMSE)	0.0024545
		Coefficient of determination (R^2^)	0.89251
		Kurtosis	3.3516
		Skewness	0.054079
Random forests	Fitting	Root mean square error (RMSE)	0.0035515
		Coefficient of determination (R^2^)	0.8158
	Prediction	Root mean square error (RMSE)	0.0035456
		Coefficient of determination (R^2^)	0.78096

## Data Availability

The data are unavailable due to privacy or ethical restrictions.
